# 24-hour urine chemistry shows higher stone formation risk after malabsorptive than restrictive type bariatric surgery

**DOI:** 10.1038/s41598-026-37440-y

**Published:** 2026-01-29

**Authors:** Alex Qinyang Liu, Eric Ka-Ho Choy, Brian Wai Hei Siu, Carol Man-Sze Lai, Steffi Kar-Kei Yuen, Ivan Ching Ho Ko, Peter Ka-Fung Chiu, Jeremy Yuen Chun Teoh, Candice Chuen-Hing Lam, Shirley Yuk-Wah Liu, Chi Fai Ng

**Affiliations:** 1 S.H. Urology Centre, Department of Surgery, Prince of Wales Hospital, The Chinese University of Hong Kong, 4/F, Lui Che Woo Clinical Sciences Building, Shatin, 999077 Hong Kong SAR China; 2 Division of Upper Gastrointestinal and Metabolic Surgery, Department of Surgery, The Chinese University of Hong Kong, 4/F, Lui Che Woo Clinical Sciences Building, Shatin, 999077 Hong Kong SAR China; 3 Li Ka Shing Institute of Health Sciences, The Chinese University of Hong Kong, Shatin, Hong Kong SAR China; 4 Center for Translational Urological Research, Shenzhen Research Institute, The Chinese University of Hong Kong, Shenzhen, 518000 China; 5 Department of Urology, Medical University of Vienna, Vienna, Austria

**Keywords:** Urolithiasis, Bariatric surgery, Renal stone, Obesity, Diseases, Medical research, Nephrology, Risk factors, Urology

## Abstract

Bariatric surgery is effective for obesity management but associated with kidney stone formation. Give the different post-operative physiology between restrictive type and malabsorptive type bariatric surgery, this study aims to compare difference in post-operative lithogenic risk profiles between these surgical types by assessing the postoperative 24-hour urine chemistry profiles. We conducted a prospective cross-sectional study of consecutive adults undergoing bariatric surgery at a tertiary center in Hong Kong between April 2017 and October 2019. A total number of 35 patients underwent malabsorptive and 55 underwent restrictive procedures. Baseline demographics, comorbidities, and postoperative 24-hour urine chemistry were assessed within 12 months after surgery. Abnormal urinary parameters were identified, with between-group comparisons performed using Mann–Whitney U and Chi-squared tests. Propensity scores were estimated using selected covariates, and stabilized inverse probability of treatment weighting (IPTW) was applied. IPTW-weighted logistic regression was used to compare the odds of abnormal urinary parameters between surgical groups. At 12 months, the malabsorptive group demonstrated significantly higher urinary oxalate and lower urinary creatinine, potassium, calcium, magnesium, citrate, urate, pH, and calcium phosphate activity compared with the restrictive group. The prevalence of hyperoxaluria (51.4% vs. 25.5%, *p* = 0.012), hypocitraturia (71.4% vs. 36.4%, *p* = 0.001), and acidic urine (54.3% vs. 20.0%, *p* = 0.001) was higher in the malabsorptive group. Conversely, hyperuricosuria was more common in restrictive patients (29.1% vs. 11.4%, *p* = 0.049). No significant differences were observed for urine volume, sodium, phosphate, or calcium oxalate activity. IPTW-weighted logistic regression demonstrated that malabsorptive procedures were associated with significantly higher odds of hyperoxaluria (OR 2.95, 95% CI 1.03–8.44), hypocitraturia (OR 4.13, 95% CI 1.40–12.21), hypomagnesuria (OR 3.26, 95% CI 1.11–9.57), and acidic urine pH (OR 3.76, 95% CI 1.33–10.64). Malabsorptive bariatric surgery is associated with more lithogenic urinary profiles than restrictive surgery, particularly hyperoxaluria, hypocitraturia, hypomagnesuria, and acidic urine, underscoring increased risk of postoperative nephrolithiasis. Close monitoring of urinary parameters and multidisciplinary management are recommended to mitigate stone risk.

## Introduction

According to the World Health Organization, over 890 million adults aged 18 years and older are living with obesity. Between 1990 and 2022, the prevalence of obesity has been more than doubled globally^1^. Obesity has been linked to various comorbidities, including type 2 diabetes, hypertension, dyslipidemia, and many more. Previous studies have shown that obese patients were more likely to form kidney stones and had increased excretion of stone-promoting substances^2,3^.

As obesity has become more prevalent over the past decades, rates of bariatric surgery have increased accordingly for its efficacy in weight loss and reduction of comorbidities related to obesity^4,5^. Bariatric surgery is considered the most effective treatment of morbid obesity^6^. Bariatric surgical procedures can be generally divided into pure restrictive procedures and procedures with malabsorptive component. The former, including but not limited to adjustable gastric banding and sleeve gastrectomy, aims at limiting the reservoir to decrease the amount of food that can be ingested. The latter, including but not limited to biliopancreatic diversion and Roux-en-Y gastric bypass, is used to limit the absorption of nutrients by bypassing predetermined length of bowels^7^, but may also incorporate restrictive principles.

Current literature has established nephrolithiasis as a known postoperative complication of bariatric surgery. Malabsorptive type surgery has been linked to the development of urinary lithogenic profiles (e.g. hyperoxaluria), increased risk of de novo kidney stone formation, recurrent stone formation, and increased stone development in patients with nephrolithiasis history^8–13^. On the contrary, several studies showed that restrictive-type surgery might improve urinary profiles and decrease the risk of nephrolithiasis in comparison to bypass surgery^14,15^. Therefore, we would like to compare the effects of malabsorptive and restrictive bariatric surgery on postoperative 24-h urine chemistry in relation to kidney stone formation.

## Methodology

This was a prospective cross-sectional study on consecutive adult patients (≥ 18 years old) who received bariatric surgery between April 2017 and October 2019 in a tertiary surgical centre in Hong Kong, China. The study was conducted in accordance with the Declaration of Helsinki. Written informed consent was obtained from all participating patients prior to study enrolment. Ethical approval was obtained before the commensal of the study, with approval granted by the Joint Chinese University of Hong Kong-New Territories East Cluster Clinical Research Ethics Committee.

All bariatric procedures were performed by two experienced bariatric surgeons, who each had performed more than 100 bariatric procedures. The standard surgical techniques used in our centre were adopted, as previously described^16,17^. Inclusion criteria are in accordance with the International Federation for the Surgery of Obesity and Metabolic Disorders—Asian Pacific Chapter Consensus Statement 2011^18^. Patients with a body mass index (BMI) ≥ 35 kg/m^2^, and patients with BMI ≥ 30 kg/m^2^ and two or more existing obesity-related comorbidities, including inadequately controlled type 2 diabetes mellitus or metabolic syndrome were eligible for bariatric surgery.

Baseline characteristics of patients, including demographics, obesity-related parameters, serum levels of biochemical markers of kidney function, incidence of hypertension, hyperlipidemia, and diabetes mellitus, were prospectively recorded at preoperative clinics. Multivitamins, “Daily one plus”, 1 tablet daily was prescribed for all patients after sleeve gastrectomy. “Daily one plus” 1 tablet daily, CaCO3 1500 mg B.D. and Rocaltrol (calcitriol) 0.5mcg daily were prescribed to patients following RYGB. These aforementioned supplements were started post-operatively once the patient can tolerate oral diet and medications during the admission for the index bariatric operation, and were continued post-operatively in the first year and beyond. 24-h urine samples were collected for chemical analysis at baseline and post-operative follow-up within 12 months. During the urine collection period, dietary restrictions were not advised. Urine volume, creatinine, sodium, potassium, calcium, magnesium, citrate, phosphate, oxalate, urate, and pH were measured and compared. Abnormal post-operative 24-h urine chemistry profile was defined according to the followings from the EAU guidelines on stone^19^: hypercalciuria (> 5 mmol/day), hyperphosphaturia (> 35 mmol/day), hyperoxalaturia (> 0.5 mmol/day), hyperuricosuria (> 4 mmol/day for female; >5 mmol/day for male), hypocitraturia (< 2.5 mmol/day), hypomagnesuria (< 3 mmol/day), and acidic pH (≤ 5.8). Ionic strengths of calcium oxalate (AP(CaOx)) and calcium phosphate (AP(CaP)) were also calculated and compared^20^.

Descriptive data were compared between the two surgical groups, with continuous variables analyzed with Mann Whitney U test, and categorical variables analyzed using Chi-squared test. Statistical significance was defined as < 0.05. Propensity scores were estimated using logistic regression using baseline covariates including age, BMI, body fat percentage, waist circumference, dyslipidaemia, and diabetes status. Stabilized inverse probability of treatment weighting was applied. Weighted comparisons of abnormal urinary parameters were performed using IPTW-weighted logistic regression, and results are reported as odds ratios with 95% confidence intervals. Data was analysed using SPSS Statistics (version 25.0. Armonk, NY: IBM Corp) and RStudio (version 2023.06.0 + 421. Posit Software).

## Results

A total of 90 patients were recruited in this study. The number of patients who received malabsorptive and restrictive bariatric surgery were 35 and 55, respectively. Table 1 summarizes the baseline characteristics of the included patients. The baseline mean age for malabsorptive and restrictive bariatric surgery were 46.5 ± 9.1 and 43.1 ± 11.3 years, respectively. Patients in the malabsorptive group had significantly lower BMI and a higher proportion of hyperlipidemia and diabetes mellitus. There was no statistically significant difference in age, gender distribution, body weight, waist circumference, serum parathyroid hormone, serum vitamin D, serum albumin, and prevalence of chronic kidney disease and hypertension between restrictive and malabsorptive groups. Among patients classified into the malabsorptive group (*n* = 35), 18 received duodeno-jejunal bypass, 16 Roux-en-Y gastric bypass, and 1 single-anastomosis gastric bypass. Among patients classified into the restrictive group (*n* = 55), 52 received laparoscopic sleeve gastrectomy, 2 laparoscopic greater curvature plication, and 1 endoscopic sleeve gastroplasty.

**Table 1:** Baseline characteristics in the 2 groups.

	Restrictive (*n* = 55)	Malabsorptive (*n* = 35)	*P* value
Age, year	43.1 ± 11.3	46.5 ± 9.1	0.137
Male sex	23 (41.8%)	21 (60.0%)	0.093
Body weight, kg	108.0 ± 18.8	103.3 ± 22.1	0.289
Waist circumference, cm	119.3 ± 12.8	117.1 ± 13.1	0.443
Fat percentage, %	37.9 ± 8.0	33.1 ± 6.8	0.005 *
Serum creatinine, µmol/L	67.4 ± 15.1	77.4 ± 24.7	0.039 *
Serum urea, mmol/L	4.9 ± 1.3	5.7 ± 1.8	0.010 *
Serum albumin, g/L	41.3 ± 3.5	39.8 ± 4.3	0.088
Hypertension	41 (74.5%)	30 (85.7%)	0.206
Hyperlipidemia	32 (58.2%)	28 (80.0%)	0.032 *
Diabetes mellitus	24 (43.6%)	29 (82.9%)	0.001 *

Continuous variables: presented as mean ± standard deviation.

Catergorical variables: presented as number (percentage).

The median follow-up period for the two groups was 12 months. Post-operative 1-year anthropometric parameters showed no statistically significant differences in terms of body weight (82.3 kg vs. 79.4 kg, *p* = 0.376) or waist circumference (100.8 cm vs. 98.8 cm, *p* = 0.260), nor did post-operative 1-year serum biochemical parameters in terms of serum creatinine (68.0 µmol/L vs. 74.5 µmol/L, *p* = 0.141), serum urea (5.2 mmol/L vs. 5.7 mmol/L, *p* = 0.238), or serum albumin (39.7 g/L vs. 38.7 g/L, *p* = 0.228). Post-operative fat percentage, however, remains higher in the restrictive group compared to the malabsorptive group (28.5% vs. 24.4%, *p* = 0.005).

The detailed post-operative 1-year 24-h urine chemistry profiles of the two groups are summarized in Table 2. The malabsorptive group had significantly lower urinary creatinine, potassium, calcium, magnesium, citrate, urate, pH, and AP(CaP) levels and significantly higher urinary oxalate levels compared to the restrictive group (see Table 2). There was no statistically significant difference in urine volume, sodium, phosphate, and AP(CaOx) level between the two groups. Table 3 summarizes the proportion of patients with abnormal urinary chemistry in the two groups. The proportion of patients with hyperoxaluria (51.4% vs. 25.5%, *p* = 0.012), hypocitraturia (71.4% vs. 36.4%, *p* = 0.001), acidic pH (54.3% vs. 20.0%, *p* = 0.001) were higher in patients receiving malabsorptive surgery than restrictive surgery. Interestingly, there was a significantly higher proportion of hyperuricosuria in the restrictive group compared to the malabsorptive group (29.1% vs. 11.4%, *p* = 0.049). No significant difference in the proportion of hypercalciuria, hyperphosphaturia, and hypomagnesuria was observed between the two groups otherwise.

**Table 2:** Post-operative 24-h urine chemistry profiles in the 2 groups.

	Restrictive (*n* = 55)	Malabsorptive (*n* = 35)	*P* value
Urine volume, L	1.5 ± 0.8	1.6 ± 0.7	0.540
Creatinine, mmol/L	9.6 ± 4.5	7.9 ± 4.6	0.103
Sodium, mmol/L	126.1 ± 45.0	110.2 ± 40.6	0.094
Potassium, mmol/L	35.9 ± 16.2	27.6 ± 12.3	0.007 *
Calcium, mmol/L	3.4 ± 1.8	2.3 ± 1.4	0.006 *
Magensium, mmol/L	3.3 ± 1.3	2.7 ± 1.4	0.062
Citrate, mmol/L	2.5 ± 1.5	1.3 ± 1.2	< 0.001 *
Phosphate, mmol/L	19.8 ± 8.8	17.2 ± 9.8	0.201
Oxalate, mmol/L	0.29 ± 0.13	0.40 ± 0.23	0.021 *
Urate, mmol/L	2.9 ± 1.4	2.4 ± 1.4	0.083
pH	6.3 ± 0.4	5.9 ± 0.4	< 0.001 *
AP(CaOx)	0.70 ± 0.49	0.66 ± 0.67	0.745
AP(CaP)	236.8 ± 561.5	49.8 ± 117.2	0.020 *

Independent t-test.

AP = ionic streng (the higher, then the higher solubility of the ionic compound).

**Table 3:** Prevalence of abnormal post-operative 24-h urine chemistry profile in the 2 groups.

	Restrictive (*n* = 55)	Malabsorptive (*n* = 35)	*P* value
Hypercalciuria (> 5mmol/day)	19 (34.5%)	6 (17.1%)	0.072
Hyperphosphaturia (> 35mmol/day)	8 (14.5%)	8 (22.9%)	0.315
Hyperoxaluria (> 0.5mmol/day)	14 (25.5%)	18 (51.4%)	0.012 *
Hyperuricosuria (> 4mmol/day for female, > 5 mmol/day for male)	16 (29.1%)	4 (11.4%)	0.049 *
Hypocitraturia (< 2.5mmol/day)	20 (36.4%)	25 (71.4%)	0.001 *
Hypomagnesuria (< 3mmol/day)	12 (21.8%)	13 (37.1%)	0.114
Acidic pH (≤ 5.8)	11 (20.0%)	19 (54.3%)	0.001 *

Propensity scores were estimated using age, BMI, body fat percentage, waist circumference, hyperlipidemia, and diabetes status. After application of stabilized inverse probability of treatment weighting, baseline covariates balance between the two groups was improved. Age, percentage body fat, hyperlipidemia achieved standardized mean differences (SMDs) below 0.1, and the rest of the covariates demonstrated SMDs below 0.25 indicating acceptable post-weighting balance. Table 4 reports the odds ratios of lithogenic abnormal 24-h urine chemistry profile after propensity score weighting. Malabsorptive procedures were consistently associated with several lithogenic urinary chemical abnormalities compared with restrictive surgery, including hyperoxaluria (OR 2.95, 95% CI 1.03–8.44; *p* = 0.044), hypocitraturia (OR 4.13, 95% CI 1.40-12.21; *p* = 0.011), hypomagnesuria (OR 3.26, 95% CI 1.11–9.57; *p* = 0.032), and acidic urine pH (OR 3.76, 95% CI 1.33–10.64; *p* = 0.013).

**Table 4:** Malabsorptive surgery’s odds ratios of abnormal 24-h urine chemistry profile after propensity score weighting.

	Odds ratio	95% confidence interval	*P* value
Hypercalciuria (> 5mmol/day)	0.31	0.10–0.99	0.049 *
Hyperphosphaturia (> 35mmol/day)	1.30	0.38–4.48	0.670
Hyperoxaluria (> 0.5mmol/day)	2.95	1.03–8.44	0.044 *
Hyperuricosuria (> 4mmol/day for female, >5mmol/day for male)	0.31	0.09–1.13	0.076
Hypocitraturia (< 2.5mmol/day)	4.13	1.40-12.21	0.011 *
Hypomagnesuria (< 3mmol/day)	3.26	1.11–9.57	0.032 *
Acidic pH (≤ 5.8)	3.76	1.33–10.64	0.013 *

## Discussion

In this study, we compared the post-operative 24-HU profile in patients receiving restrictive type and malabsorptive type bariatric surgery. Our study suggested that malabsorptive type bariatric surgery was more prone to the development of post-operative lithogenic urinary profile than restrictive type.

Malabsorptive type of bariatric surgery, for example, Roux-en-Y gastric bypass and biliopancreatic diversion, causes weight loss by reducing gastric reservoir and small intestine bypass, which decreases oral calorie intake and absorptive surface area^21^. Restrictive types of bariatric surgery, such as sleeve gastrectomy and gastric banding, cause weight loss by surgically reducing gastric volume and decreasing oral food intake. Sleeve gastrectomy and Roux-en-Y gastric bypass are the most common bariatric surgical procedures^22^. Bariatric surgery can treat severe obesity effectively^6^. However, it has been associated with an increased risk of postoperative urolithiasis^23^.

At postoperative follow-up, our data demonstrated that patients who underwent malabsorptive type surgery had worse lithogenic 24-HU chemistry profiles than those receiving restrictive surgery. Significantly higher oxalate (0.40 mmol/L vs. 0.29 mmol/L, *p* = 0.025), lower creatinine (7.9 mmol/L vs. 9.6 mmol/L, *p* = 0.043), potassium (27.6 mmol/L vs. 35.9 mmol/L), calcium (2.3 mmol/L vs. 3.4 mmol/L, *p* = 0.002), magnesium (2.7 mmol/L vs. 3.3 mmol/L, urate (2.4 mmol/L vs. 2.9 mmol/L), citrate (1.3 mmol/L vs. 2.5 mmol/L, *p* < 0.001), urine pH (5.9 vs. 6.3, *p* < 0.001) and ionic activity of calcium phosphate (49.8 vs. 236.8, < 0.001) were observed in the malabsorptive group. Patients receiving malabsorptive type bariatric surgery also had a significantly higher proportion of postoperative hyperoxaluria (51.4% vs. 25.5%, *p* = 0.012), hypocitraturia (43.4% vs. 18.5%, *p* = 0.015), acidic pH (54.3% vs. 20%, *p* = 0.001) and lower proportion of hyperuricosuria (11.4% vs. 29.1%) than those in the restrictive group. These trends were consistently observed even after adjustment for baseline differences using stabilized inverse probability of treatment weighting. In IPTW-weighted logistic regression analyses, malabsorptive surgery was associated with statistically significant higher odds of hyperoxaluria (OR 2.95, 95% CI 1.03–8.44; *p* = 0.044), hypocitraturia (OR 4.13, 95% CI 1.40-12.21; *p* = 0.011), hypomagnesuria (OR 3.26, 95% CI 1.11–9.57; *p* = 0.032), and acidic urine pH (OR 3.76, 95% CI 1.33–10.64; *p* = 0.013)—all of which lithogenic risk factors. Conversely, the odds of hypercalciuria were significantly lower following malabsorptive surgery (OR 0.31, 95% CI 0.10–0.99) as enteric calcium and Vitamin D absorption is affected after bypass.

Dietary factors and intestinal changes can elucidate post-surgical hyperoxaluria in malabsorptive surgery. For dietary factors, intestinal oxalate absorption is determined by the formation of calcium oxalate complexes. Oxalate-rich or calcium-poor diet decreases luminal calcium oxalate complexes, which are poorly soluble and nonabsorbable, resulting in increased free oxalate absorption. Vitamin C supplements, which are usually indicated in post-surgical management, may contribute to hyperoxaluria as it is metabolized to oxalate in our body. For intestinal changes, malabsorption of lipids causes intraluminal accumulation of free fatty acids. These unabsorbed fatty acids tend to bind dietary calcium and reduce the amount of calcium available to bind oxalate, leading to increased free oxalate absorption from dietary intake^24^.

Furthermore, increased intestinal exposure to bile acid and fatty acids might precipitate microbiota change and decreased colonization by oxalate-degrading bacteria, e.g. *Oxalobacter formigenes*, and enhanced luminal permeability of oxalate absorption in colon, all of which lead to hyperoxaluria^25^. Lastly, a higher prevalence of diabetes mellitus and hyperlipidaemia in the malabsorptive group may also contribute to hyperoxaluria. Diabetes mellitus can increase plasma levels of oxalate precursors, e.g. glyoxal and glyoxylate, hence oxalate production and hyperoxaluria. As a result, patients with hyperlipidaemia, especially high total triglyceride, are associated with higher urinary oxalate excretion^26^.

In malabsorptive surgeries, significant loss of bicarbonate ion from chronic diarrhea may contribute to metabolic acidosis. Subsequent increase in acid load stimulates renal tubular reabsorption mediated by sodium-dicarboxylate cotransporter-1 and increases citrate metabolism within renal cells mediated by ATP-citrate lyase, resulting in hypocitraturia.

^27,28^. Also, diabetes mellitus-associated insulin resistance is linked with hypocitraturia, possibly due to defective renal ammonium production and changes in renal tubules’ sodium-potassium and hydrogen transport mechanism. Hyperlipidemic patients might exhibit insulin resistance, which similarly impairs renal ammoniogenesis, and systemic acidosis, which results in increased renal citrate reabsorption^29,30^. Metabolic acidosis from chronic diarrhoea secondary to the bypass surgery may also lead to the low urine pH found in the malabsorptive group as increased renal excretion of hydrogen ions and reabsorption of bicarbonate ions occur. Diabetes mellitus and hyperlipidemia also contribute to increased urine acidity through metabolic acidosis and increased net acid excretion^31^.

A decreased urinary creatinine excretion can be explained by reduced muscle mass after bariatric surgery^32,33^. Lower creatinine levels in the malabsorptive group may be linked to greater disturbance of protein absorption as the stomach is usually bypassed rather than only resected, as indicated in restrictive surgeries. The majority of potassium, calcium, magnesium and purine is absorbed in the duodenum and jejunum. Malabsorptive surgeries reduce the surface area for potassium, calcium, magnesium, and purine absorption by bypassing the duodenum and part of the jejunum, thus leading to lower post-surgical urinary potassium, calcium, magnesium and urate^34,35^. Restrictive surgeries impose less disturbance on potassium absorption as it does not affect the surface area for absorption.

AP (CaP) represents the product of the concentration of calcium ions and phosphate ions. In our cohort, the malabsorptive group had significantly lower urinary calcium (*P* = 0.002) and lower urinary phosphate (*P* > 0.05). Lower urinary calcium (& phosphate) ions decrease the supersaturation of calcium phosphate salt in urine, resulting in decreased calcium phosphate crystallisation. More acidic urine pH can interfere with the conversion of hydrogen diphosphate to hydrogen monophosphate, which decreases calcium phosphate stone formation^36^. However, as citrate is a known inhibitor of kidney stone formation, it remains unsure how the interplay among low urinary calcium ion, acidic urine pH and hypocitraturia have an overall suppressive effect over ionic activity of urinary calcium phosphate, and hence lower risk of precipitation in malabsorptive bariatric surgeries than restrictive surgeries.

Our findings are largely compatible with previous literature on post-operative 24-h urine changes after bariatric surgery^8–11,13,37–40^. Park et al. and Valezi et al. reported postoperative hyperoxaluria, hypocitraturia and low urine volume in Roux-en-Y gastric bypass surgery^9,39^. Semins et al. and Penniston et al. found a higher proportion of postoperative hyperoxaluria and hypocitraturia in nephrolithiasis-naïve patients following malabsorptive surgery than restrictive surgery^38,41^. Similar effects of malabsorptive surgery and restrictive surgery on lithogenic urinary profiles were also observed in patients with nephrolithiasis history^13^. Our data did not show a statistically significant difference in urine volume between groups.

However, we found a significant decrease in urinary pH in patients undergoing malabsorptive surgery than restrictive surgery, which was seldom reported in previous studies. One of the interesting findings was that significantly higher post-operative hyperuricosuria was observed in patients undergoing restrictive surgery. The higher prevalence of hyperuricosuria in restrictive surgery may be explained by high dietary protein intake and less-impaired post-surgical purine absorption^42^. It has also been hypothesized that reduced renin-angiotensin-aldosterone system (RAAS) activation after metabolic surgery may reduce proximal tubular reabsorption of uric acid, thus promoting hyperuricosuria^43^. This will, in turn, increase the risk of uric acid stone formation^44,45^. In addition, the higher ionic activity product of calcium phosphate observed in the restrictive group would also implicate a higher risk of calcium phosphate super-saturation and crystallization. Therefore, further studies on the risk of uric acid and calcium phosphate stone formation in patients receiving restrictive-type surgery are warranted.

There are several limitations in this study. First, no preoperative urine chemistry was saved; therefore, we could not evaluate any changes in urinary profiles longitudinally. Secondly, there could be inherent bias due to study design, e.g. selection bias, given the observational nature of this study. Admittedly, there were unbalanced baseline characteristics in the two groups. Thirdly, we did not obtain the post-operative data at a fixed time point, e.g. one year. Post-operative urine samples were saved from post-op six months to 12 months, and the median time from surgery to urine sample collection between the two groups is the same, i.e. 12 months. Lastly, there is an absence of systematic radiological assessment for nephrolithiasis, such as preoperative or postoperative CT imaging. While patients were regularly reviewed during postoperative follow-up and stone-related symptoms would have been reported if present, the study was not designed to formally capture symptomatic stone events as a predefined outcome. No clinically apparent stone-related pain episodes were documented during follow-up; however, asymptomatic or subclinical stone formation could not be assessed in the absence of routine imaging. Therefore, the findings of this study should be interpreted as reflecting differences in lithogenic urinary risk profiles rather than confirmed stone formation. Longitudinal studies incorporating standardized imaging surveillance and prospective recording of stone-related clinical events are warranted.

In conclusion, malabsorptive type bariatric surgery was associated with the development of hyperoxaluria, hypocitraturia, and low urine pH for urinary calculi formation than restrictive type surgery. Post-operative monitoring of urinary profiles, dietary advice, symptomology, and radiological findings would be helpful in preventing stone formation and identifying stone disease in these patients. Multi-disciplinary communication between urologists and metabolic surgeons is recommended.

## Data Availability

The datasets are not publicly available due to patient privacy, but de-identified data can be available from the corresponding authors upon reasonable request and with the permission of the institutional ethics committee.
